# Editorial: Brain Plasticity and Contribution of the Emotional Brain to Neural Remodelling After Injury

**DOI:** 10.3389/fneur.2020.606271

**Published:** 2020-10-26

**Authors:** Manuel Gaviria, Alessia Celeghin, Adina T. Michael-Titus, Patrick N. Pallier

**Affiliations:** ^1^Clinical Neurosciences, Institut Equiphoria/Alliance Equiphoria, La Canourgue, France; ^2^Department of Psychology, University of Turin, Turin, Italy; ^3^Centre for Neuroscience, Surgery and Trauma, Blizard Institute, Barts and The London School of Medicine and Dentistry, London, United Kingdom

**Keywords:** neurological diseases, long-term disability, neuroplasticity, emotional brain, motivation, neurorehabilitaiton, functional recovery

In our current neurorehabilitation practice, we are sometimes faced with patients pushing themselves beyond their limits, an attitude that may have a substantial impact on their functional abilities. This “state of mind” is often present when relevant and enjoyable activities are performed, allowing individuals to experience powerful sensory, motor, cognitive, and social episodes. A common feature underlying these events is most likely the capacity to evoke and feel emotions, thus modifying or influencing the neuroendocrine and autonomic nervous system and enhancing cognitive and overall brain functioning by engaging multiple cortical and subcortical structures. Therefore, a crucial question is whether motivation and emotions are key actors in reshaping neural networks after central nervous system (CNS) injury. This Research Topic aims to gather observations on innovative approaches that focus on the impact of emotional and motivational networks on functional capacities in major neurological disorders. It includes 2 original articles, 2 reviews, and 1 case report that relate to the contribution of emotions and motivation to neuroplasticity and functional recovery.

Whether developmental, post-traumatic or degenerative, major neurological diseases lead to long-term substantial disability that stems mainly from cognitive and psychomotor impairment, with dramatic emotional and psychosocial consequences. Clinical management of severe disabilities is often ineffective; when beneficial, the mechanisms of action leading to the favorable outcome are to a large extent unclear. Current theories emphasize the importance of the brain connectome in functional recovery, through neuroplasticity. The level of impairment of disabled individuals may reflect large-scale disturbances of neural networks that result from damage of connections between major brain hubs, and this could determine the clinical outcome. In the context of functional recovery, how perception, emotion, motivation, and cognition interact and lead to an improved outcome, and how the activity of brain regions involved in such diverse domains is coordinated, are questions that remain to be resolved.

Under physiological conditions, emotional and motivational processing shapes brain responses by increasing functional connections across dissimilar brain hubs and regions. It has been hypothesized that such interactions lead to improved behavioral performance during challenging tasks, and that networks can be considered as dynamic processes whose evolution is closely tied to the underlying mechanisms supporting behavior. The dynamic view of brain functioning ascribes to emotion substantial influences on information processing, in a way that goes beyond the conventional boundaries differentiating emotion, cognition, and action (1, 2).

Apart from studies conducted in individuals suffering from mental disorders associated with abnormal emotional regulation, such as schizophrenia, major depression, bipolar disorder, post-traumatic stress disorder, anxiety and panic disorders, few have explored the effects of emotions in conditions such as neurotrauma or neurodevelopmental disorders, which affect the cognitive and/or sensorimotor domains. One of the fundamental properties of the CNS is the ability to adapt its response to a wide range of complex changes, including those generated by an injury. This inherent plasticity promotes at least some functional recovery. However, spontaneous mechanisms of repair are rarely sufficient to support significant long-term recovery, while post-injury experience can be a powerful modulator of functional recovery. In recent years, it has become evident that environmental enrichment has strong effects on neuroplasticity and neurological improvement. Its beneficial effects have been demonstrated in a wide variety of experimental models of brain disorders, and include cognitive improvement, delayed onset of neurodegenerative disease, enhanced cellular and molecular plasticity, and stimulation of the synthesis of neuromodulators that affect the levels of arousal, motivation, attention, affection and emotion of an individual (such as noradrenaline, acetylcholine, dopamine, or serotonin). These endogenous molecules are strongly involved in the induction and maintenance of synaptic plasticity at a molecular, anatomical, and functional level. Experiences that are most relevant to the individual, and are also more intense and frequent, are likely to produce much faster and profound brain remodeling (3, 4). In this context, the concept of emotional intelligence, as defined by the Bar-On's mixed model, is highly relevant. Its four components—emotional awareness, emotional recognition, empathy/prosocial behavior, and emotional memory, to which Goleman had initially included motivation (5), seem intuitively paramount from a neurorehabilitation perspective (6). If it can be proved that such a powerful driver for brain plasticity is meaningful and can be stimulated, then practitioners should harness it for an improved neurorehabilitation regime.

The current collection proposes some pivotal ideas that are in line with the involvement of the emotional dimension in neuroplasticity and functional recovery. A first paper, by Wang et al., highlights the importance of an enriched environment—not just recreational tasks, but also novelty and social stimuli from mates and healthy individuals, in the promotion of cognitive function, after cerebral ischemia in a permanent middle cerebral artery occlusion mouse model. They show that cognitive function is supported via bilateral synaptic remodeling, with the contribution of the contralateral brain. In a second paper, Shibuki et al. demonstrate how affective stimulation by alarming visual stimuli can help the long-term improvement of conscious sight recovery from cortical blindness, via intact functional connections between the superior colliculus and the amygdala, to the higher visual cortex. The paper by Ajina et al. reveals the importance of these same residual structures and functions in the processing of subtle and complex facial expressions (such as trustworthiness and dominance) following cortical blindness, showing that socially salient but emotionally neutral facial expressions survive through the same underlying mechanism as affective blindsight. The paper by Yang and Chang discusses the evidence of efficacy of repetitive transcranial magnetic stimulation on pain management and suggests that one's emotional state should also be taken into consideration to improve treatment efficacy in chronic pain conditions. Finally, the article by Duffell and de Neufvillle Donaldson highlights the importance of motivation in rehabilitation. The authors demonstrate that more effective neuroplasticity and faster locomotor recovery could be achieved after spinal cord injury, through repetitive training using functional electrical stimulation associated with the use of virtual reality and biofeedback, to motivate the patients and encourage supraspinal drive, which may otherwise be lacking. This pioneering work opens up novel avenues for research in neurorehabilitation. Since our current therapeutic arsenal for neurorehabilitation is rather limited and inefficient, novel strategies to replace or complement current methods are urgently needed.

## Author Contributions

MG: conceptualization, methodology, data curation, writing—original draft preparation, supervision, and project administration. AC, AM-T, PP, and MG: validation, investigation, and writing—review and editing. All authors have revised the paper critically for important intellectual content, approved the final version and agree to be accountable for all aspects of the work in ensuring that questions related to the accuracy or integrity of any part of the work are appropriately investigated and resolved.

## Conflict of Interest

The authors declare that the research was conducted in the absence of any commercial or financial relationships that could be construed as a potential conflict of interest.

## References

[1] Pessoa L. A network model of the emotional brain. Trends Cogn Sci. (2017) 21:357–71. 10.1016/j.tics.2017.03.002 28363681 PMC5534266

[2] Pessoa L McMenamin B. Dynamic networks in the emotional brain. Neuroscientist. (2017) 23:383–96. 10.1177/1073858416671936 27784761 PMC5405011

[3] Kolb B Muhammad A. Harnessing the power of neuroplasticity for intervention. Front Hum Neurosci. (2014) 8:377. 10.3389/fnhum.2014.00377 25018713 PMC4072970

[4] Sale A Berardi N Maffei L. Environment and brain plasticity: towards an endogenous pharmacotherapy. Physiol Rev. (2014) 94:189–234. 10.1152/physrev.00036.2012 24382886

[5] Goleman D. What makes a leader? Harvard Bus Rev. (1998) 76:93–102. 10187249

[6] Hogeveen J Salvi C Grafman J. “Emotional intelligence”: lessons from lesions. Trends Neurosci. (2016) 39:694–705. 10.1016/j.tins.2016.08.007 27647325 PMC5807001

